# Oxidative Damage, Antioxidant Capacity, and Apoptotic Activation in Varicocele: Biochemical Evidence of Improvement After Surgical Repair

**DOI:** 10.3390/antiox15040455

**Published:** 2026-04-05

**Authors:** Erdem Orman, Hakki Uzun, Merve Huner Yigit, Ertugrul Yigit, Huseyin Cinar Zihni, Gorkem Akca

**Affiliations:** 1 Department of Urology, Faculty of Medicine, Recep Tayyip Erdogan University, Rize 53100, Türkiye; erdem.orman@erdogan.edu.tr (E.O.); gorkem.akca@erdogan.edu.tr (G.A.); 2 Department of Medical Biochemistry, Faculty of Medicine, Recep Tayyip Erdogan University, Rize 53100, Türkiye; merve.huner@erdogan.edu.tr; 3 Department of Medical Biochemistry, Faculty of Medicine, Karadeniz Technical University, Trabzon 61080, Türkiye; ertugrulyigit@ktu.edu.tr; 4 Department of Medical Biochemistry, Graduate School of Health Sciences, Karadeniz Technical University, Trabzon 61080, Türkiye; czihni18@gmail.com

**Keywords:** varicocele, male infertility, oxidative stress, varicocelectomy, seminal plasma

## Abstract

To evaluate seminal oxidative stress, antioxidant defense, apoptosis-related activity, and Sertoli cell biomarkers in infertile men with grade 3 varicocele versus normozoospermic controls, and to assess postoperative changes after varicocelectomy. This prospective observational case–control study included 39 infertile men with grade 3 clinical varicocele and 44 normozoospermic controls. Seminal plasma levels of Malondialdehyde (MDA), 8-hydroxy-2′-deoxyguanosine (8-OHdG), superoxide dismutase (SOD), glutathione peroxidase-1 (GPx-1), reduced glutathione (GSH), nuclear factor erythroid 2–related factor 2 (NRF2), Kelch-like ECH-associated protein 1 (KEAP1), caspase-3, anti-Müllerian hormone (AMH), and inhibin B were measured by ELISA. Testicular volume, semen parameters, and diagnostic performance were also evaluated. Compared with controls, patients with varicocele had lower testicular volumes and impaired semen parameters. Seminal 8-OHdG and caspase-3 levels were higher, whereas SOD and inhibin B levels were lower. Baseline MDA, GPx-1, GSH, NRF2, KEAP1, and AMH levels did not differ significantly. After varicocelectomy, sperm concentration, total sperm count, progressive and total motility, total motile sperm count, morphology, and round cell count improved significantly. Postoperatively, caspase-3, MDA, and KEAP1 decreased, whereas SOD, GPx-1, GSH, NRF2, and inhibin B increased significantly. 8-OHdG showed a borderline decrease, and AMH remained unchanged. SOD showed the best diagnostic performance. Grade 3 varicocele is associated with oxidative DNA damage, impaired antioxidant defense, increased apoptotic signaling, and altered Sertoli cell-related seminal biomarkers. Varicocelectomy partially restores redox homeostasis, which may contribute to improved spermatogenic function.

## 1. Introduction

Male infertility represents a significant global health concern, affecting a substantial proportion of couples worldwide [1]. Infertility affects approximately 8–12% of couples of reproductive age, with male factors accounting for nearly half of all cases [2]. Among the identifiable causes, varicocele is the most common surgically correctable condition [3]. Varicocele, characterized as abnormal dilation of veins of the pampiniform plexus, is detected in approximately 15% of the general male population, 35–40% of men with primary infertility, and up to 80% of those with secondary infertility [4]. Despite its high prevalence and well-established association with impaired semen quality, the molecular basis of varicocele-induced testicular dysfunction remains incompletely elucidated [5].

Increasing evidence suggests that oxidative stress plays a central role in the pathophysiology of varicocele-associated infertility [3,6,7]. Elevated scrotal temperature, venous stasis, hypoxia, and inflammatory activation contribute to excessive production of reactive oxygen species (ROS) [8]. When antioxidant defense mechanisms are insufficient to neutralize ROS, redox imbalance occurs, leading to lipid peroxidation and oxidative DNA damage [9,10]. Malondialdehyde (MDA) and 8-hydroxy-2′-deoxyguanosine (8-OHdG) are widely accepted biomarkers reflecting lipid peroxidation and oxidative DNA injury, respectively [11,12].

Beyond direct structural damage, persistent oxidative stress may trigger intrinsic apoptotic pathways. ROS-induced mitochondrial dysfunction facilitates activation of caspase-3, a key regulator of apoptosis [13,14]. Increased apoptotic activity has been demonstrated in varicocele, affecting germ cells as well as supportive cell populations essential for spermatogenesis, including Sertoli and Leydig cells [15,16,17]. In addition, the NRF2–KEAP1 signaling pathway represents a central regulator of cellular redox homeostasis and suppresses caspase-mediated apoptotic pathways [18]. These findings suggest that oxidative stress and apoptosis may represent interconnected mechanisms contributing to impaired spermatogenesis.

Varicocelectomy has been associated with improvements in semen parameters, reductions in oxidative burden, and decreased sperm DNA fragmentation [19,20,21]. However, whether surgical correction restores redox balance and apoptotic signaling in a coordinated manner remains unclear. Moreover, data simultaneously evaluating oxidative stress markers, antioxidant capacity, apoptotic mediators and NRF2 -KEAP1 molecular pathway in comparison with normozoospermic men and following varicocelectomy are limited.

Therefore, the present study aimed to (i) compare seminal redox homeostasis parameters, antioxidant status, caspase-3-mediated apoptotic activity, and molecular pathways between infertile men with varicocele and normozoospermic controls, and (ii) evaluate whether varicocelectomy partially restores this molecular imbalance.

## 2. Materials and Methods

### 2.1. Study Design and Participants

This prospective case–control observational study was conducted at the Andrology Unit, Faculty of Medicine, Recep Tayyip Erdoğan University, between June 2023 and September 2025. The study protocol was approved by the Clinical Research Ethics Committee of Recep Tayyip Erdoğan University (Decision No: 2023/267), and written informed consent was obtained from all participants prior to enrollment. The study included infertile men diagnosed with grade 3 clinical varicocele confirmed by physical examination and scrotal Doppler ultrasound (CDUS) and age-comparable normozoospermic controls. Additionally, a longitudinal pre–post analysis was performed in the varicocele group following varicocelectomy. Eligible participants were men aged 18–45 years who had at least two semen analyses, documented sex steroid hormone profiles, and scrotal CDUS. Exclusion criteria included active smoking, regular alcohol consumption, secondary infertility, prior pelvic or inguinoscrotal surgery, endocrine disorders, history of genital infection, use of antioxidant or fertility-related medications within the previous three months, and chronic systemic diseases.

Normozoospermic control group: Men with no clinical or CDUS-detected varicocele and with semen parameters within the normal reference ranges according to WHO 6th edition criteria [22]. Varicocele group: Patients diagnosed with Grade 3 clinical varicocele and oligo-astheno-teratospermia (OAT) confirmed by physical examination and CDUS. Patients in the varicocele group underwent microsurgical subinguinal varicocelectomy and were re-evaluated 6 months postoperatively. This follow-up interval was selected because spermatogenesis requires approximately 74 days to complete, and an additional 12–14 days are needed for epididymal sperm maturation and transit, totaling roughly 90 days per spermatogenic cycle. A 6-month follow-up therefore encompasses two complete spermatogenic cycles, which is consistent with current guideline recommendations and published evidence indicating that the majority of semen parameter improvements following varicocelectomy are observed within this period [23,24].

### 2.2. Clinical Evaluation and Varicocele Diagnosis

All physical examinations were performed by the same experienced urologist under standardized room temperature conditions with the patient in the standing position. Varicocele grading was based on the Dubin and Amelar classification, and only Grade 3 cases were included to ensure homogeneity of disease severity [25]. Testicular volume was calculated using the Lambert formula: V = L × W × H × 0.71 [26]. On CDUS, a pampiniform plexus vein diameter ≥ 3 mm and venous reflux ≥ 2 s during Valsalva maneuver were considered diagnostic [27].

### 2.3. Hormonal and Biochemical Analysis

Venous blood samples were collected between 09:00 and 11:00 AM after overnight fasting. Serum FSH, LH, total testosterone (tT), and estradiol (E2) were measured using chemiluminescent microparticle immunoassay (Siemens ADVIA Centaur XPT, Erlangen, Germany). Metabolic parameters including fasting glucose and lipid profile were analyzed using an automated chemistry analyzer (Beckman Coulter AU5800, Brea, CA, USA).

### 2.4. Semen Analysis and Seminal Plasma Preparation

Semen samples were obtained after a standardized abstinence period of 3 days, as per laboratory protocol. Although the WHO 6th edition recommends an abstinence period of 2–7 days for routine semen analysis, a uniform 3-day abstinence was applied to all participants in order to minimize inter-individual variability in seminal oxidative stress parameters attributable to differences in ejaculatory abstinence duration [22]. Seminal volume, sperm concentration (n/mL) and total sperm count was measured. Progressive and total sperm motility assessment was performed manually under phase-contrast microscopy (BX43 microscope (Olympus, Tokyo, Japan), (400× magnification). After routine analysis, samples were centrifuged at 3000× g for 10 min obtain by seminal plasma. In addition, seminal pH, viscosity, and number of round cells was measured. Seminal plasma was separated, aliquoted, and stored at −80 °C until biochemical analysis.

### 2.5. Assessment of Redox Homeostasis, Apoptotic Activity, and Sertoli Cell Function

Seminal plasma levels of oxidative damage markers (MDA: E-EL-0060, and 8-OHdG: E-EL-0028), antioxidant defense markers (superoxide dismutase (SOD): E-EL-H1113, glutathione peroxidase-1 (GPx-1): E-EL-H5410, and reduced glutathione (GSH): E-EL-0026), redox-regulatory pathway components (NRF2: E-EL-H1564, and KEAP1: E5534Hu), an apoptosis-related marker (caspase-3: E-EL-H6282), and Sertoli cell function markers (anti-Müllerian hormone (AMH): E-EL-H6234, and inhibin-B: E-EL-H0313) were measured using commercially available enzyme-linked immunosorbent assay (ELISA) kits (Elabscience, Houston, TX, USA and BT LAB, Shanghai, China) in accordance with the manufacturers’ instructions. All assays were performed in duplicate. Absorbance was read at 450 nm, and concentrations were calculated using standard calibration curves. Total protein concentration in seminal plasma was determined using the Pierce BCA Protein Assay Kit (Thermo Fisher Scientific, Rockford, IL, USA) according to the manufacturer’s instructions, with bovine serum albumin (BSA) used as the standard. Biomarker concentrations obtained by ELISA were normalized to total protein content and expressed as pg/mg protein or ng/mg protein, as appropriate, to account for inter-sample variability in seminal plasma protein concentration. The oxidative–antioxidant–apoptotic axis was conceptualized as an integrated molecular pathway to evaluate coordinated changes before and after varicocelectomy. As measurements were performed in acellular seminal plasma using ELISA, this approach reflects biomarker associations consistent with pathway involvement rather than direct verification of intracellular molecular interactions.

### 2.6. Statistical Analysis

All analyses were performed using OriginPro 2025 (OriginLab, Northampton, MA, USA). Normality of continuous variables was assessed using the Shapiro–Wilk test together with visual inspection of histograms and Q–Q plots. Data are presented as mean ± SD for approximately normally distributed variables and as median (Q1–Q3) for non-normally distributed variables. For between-group comparisons (normozoospermic controls vs. varicocele), the independent-samples *t* test was used for normally distributed variables; otherwise, the Mann–Whitney U test was applied. The between-group difference in sample size was modest (44 vs. 39) and was therefore not considered sufficient to materially affect the validity of the selected parametric or non-parametric comparisons. For within-subject comparisons (pre- vs. post-varicocelectomy), the paired *t* test was used for normally distributed variables, whereas the Wilcoxon signed-rank test was applied for non-normally distributed variables (two-tailed). The diagnostic performance of seminal biomarkers was evaluated using receiver operating characteristic (ROC) curve analysis. The area under the curve (AUC) with 95% confidence intervals (CI) was calculated. The optimal cut-off was defined by the Youden index [28], and the corresponding sensitivity and specificity were reported. Because biomarkers differed in directionality, ROC analyses were constructed to match the expected biological pattern: for biomarkers increased in varicocele (e.g., caspase-3 and 8-OHdG), higher values were defined as the positive state; for biomarkers decreased in varicocele (e.g., SOD and inhibin B), the positive state was defined by lower values (“smaller test values = more positive”) to avoid inverted AUCs and to ensure consistent clinical interpretation. ROC results were therefore presented in separate panels for increasing and decreasing biomarkers. Associations between postoperative percent changes (Δ%) in seminal ELISA biomarkers and changes in reproductive parameters were assessed using Spearman’s rank correlation. All tests were two-tailed, and *p* < 0.05 was considered statistically significant.

## 3. Results

### 3.1. Baseline Demographic and Clinical Characteristics

Baseline demographic and clinical characteristics of the normozoospermic control group and patients with varicocele are summarized in Table 1. All routine clinical and laboratory variables—including BMI, lipid profile, fasting glucose, creatinine, C-reactive protein, and hematologic parameters—were comparable between the two groups (*p* > 0.05).

The varicocele group was slightly younger than the normozoospermic control group (median 28 vs. 29 years; *p* = 0.021) and had a modestly lower waist circumference (median 88 vs. 95 cm; *p* = 0.049). In contrast, mean height was slightly higher in the varicocele group (178.7 ± 6.01 vs. 175.5 ± 6.49 cm; *p* = 0.025). No other significant demographic or metabolic differences were observed.

### 3.2. Baseline Reproductive Hormones, Testicular Volume, and Semen Parameters

Reproductive hormone levels, testicular volume measurements, and semen characteristics are presented in Table 2. Patients with varicocele exhibited markedly reduced testicular volumes compared with normozoospermic controls, including right, left, and total testicular volume (all *p* < 0.001). In contrast, serum total testosterone and estradiol levels did not differ significantly between groups (*p* > 0.05).

Semen quality was substantially impaired in the varicocele group. Sperm concentration, total sperm count, and total motile sperm count were significantly lower than the normozoospermic controls (all *p* < 0.001). Similarly, total motility, progressive motility (rapid and slow progressive fractions), and normal morphology were significantly reduced (all *p* ≤ 0.001). Liquefaction time was slightly shorter in the varicocele group (*p* = 0.049), whereas semen volume and pH were comparable between groups.

### 3.3. Seminal Oxidative Stress, Antioxidant Defense, Sertoli Cell Markers, and Apoptosis

**Figure 1:**
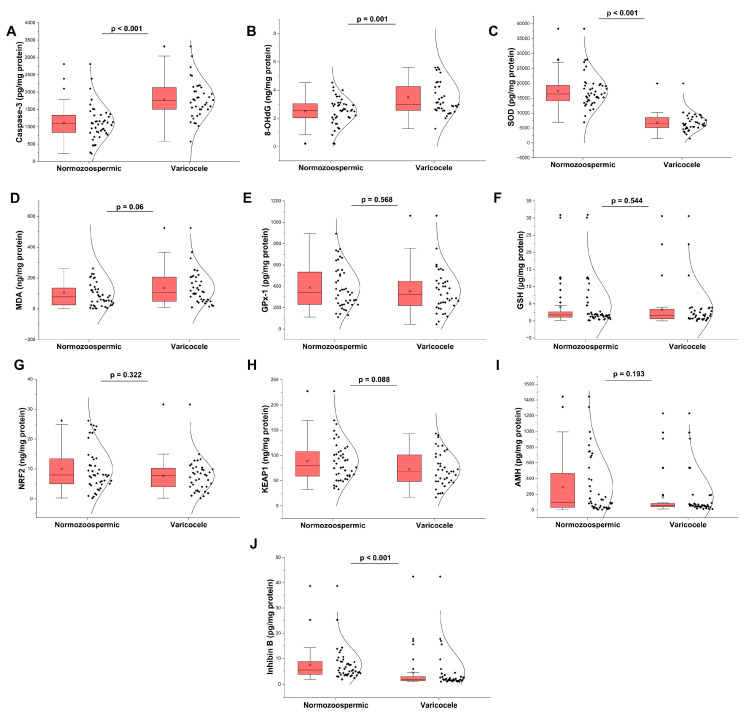
Seminal oxidative stress/antioxidant status, Sertoli cell markers, and apoptosis in normozoospermic controls and varicocele patients. Violin–box plots showing seminal levels of (**A**) Caspase-3, (**B**) 8-OhdG, (**C**) SOD, (**D**) MDA, (**E**) GPx-1, (**F**) GSH, (**G**) NRF2, (**H**) KEAP1, (**I**) AMH, and (**J**) Inhibin B in normozoospermic controls (n = 44) and varicocele patients (n = 39). Individual dots represent participants; boxes show median and IQR, and violins depict the distribution. Between-group comparisons were performed using the Mann–Whitney U test; *p* values are indicated above each panel.

Seminal biomarker profiles are illustrated in Figure 1. Compared with normozoospermic controls, patients with varicocele demonstrated significantly elevated levels of 8-OHdG and caspase-3 (both *p* ≤ 0.001). In parallel, the antioxidant enzyme SOD was markedly reduced in the varicocele group (*p* < 0.001). Regarding Sertoli cell function, inhibin B levels were significantly lower in varicocele patients (*p* < 0.001), whereas AMH levels did not differ between groups (*p* > 0.05). No statistically significant differences were observed for MDA, GPx-1, GSH, NRF2, or KEAP1, although MDA and KEAP1 showed borderline trends.

### 3.4. Diagnostic Performance of Seminal Biomarkers

**Figure 2:**
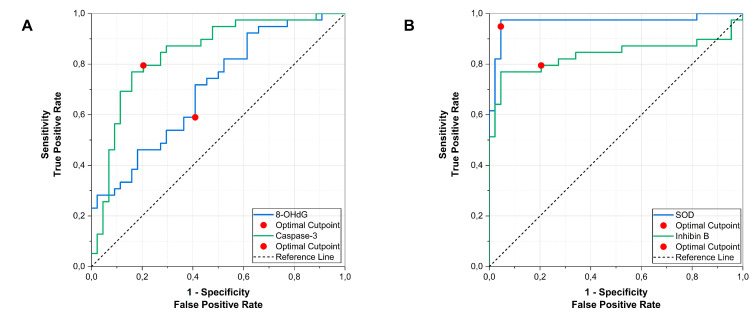
ROC curve analysis of seminal biomarkers for discriminating varicocele patients from normozoospermic controls. Receiver operating characteristic (ROC) curves evaluating the ability of seminal biomarkers to distinguish varicocele (n = 39) from normozoospermic controls (n = 44). (**A**) Increasing biomarkers (caspase-3 and 8-OHdG; higher values indicate the positive state). (**B**) Decreasing biomarkers (SOD and inhibin B; ROC constructed with lower values indicating the positive state). The diagonal line denotes the reference (no-discrimination) line; marked points indicate the optimal cut-off.

Roc analyses were done to evaluate the diagnostic performance of the biomarkers. Among biomarkers elevated in varicocele, caspase-3 demonstrated good discriminatory performance (AUC 0.843, 95% CI 0.755–0.930; *p* < 0.001). The optimal cut-off value was ≥1368.3 pg/mg protein, corresponding to 79.5% sensitivity and 79.5% specificity. 8-OHdG showed moderate diagnostic accuracy (AUC 0.704, 95% CI 0.593–0.815; *p* = 0.001), with an optimal cut-off of ≥2.87 ng/mg protein (Figure 2A).

Among biomarkers reduced in varicocele, SOD showed excellent discriminatory ability (AUC 0.967, 95% CI 0.923–1.000; *p* < 0.001), with an optimal cut-off of ≤10,052.4 pg/mg protein (sensitivity 94.9%, specificity 95.5%). Inhibin B also demonstrated good performance (AUC 0.837, 95% CI 0.735–0.939; *p* < 0.001), with an optimal cut-off of ≤4.03 pg/mg protein (Figure 2B).

### 3.5. Changes from Baseline to Postoperative Assessment

Preoperative and postoperative hormonal and semen parameters are presented in Table 3. Gonadotropins and sex steroids (including FSH, LH, estradiol, and total testosterone) did not change significantly following surgery (all *p* > 0.05). In contrast, semen quality improved markedly after surgery. Significant increases were observed in sperm concentration, total sperm count, rapid and slow progressive motility, total motility, total motile sperm count (TMSC), and morphology (all *p* < 0.001). Additionally, round cell counts decreased significantly after surgery (*p* < 0.001). Semen volume, pH, liquefaction time, viscosity, and non-progressive motility remained largely unchanged.

### 3.6. Changes in Seminal Oxidative Stress, Antioxidant Defense, Sertoli Cell Markers, and Apoptosis After Varicocelectomy

**Figure 3:**
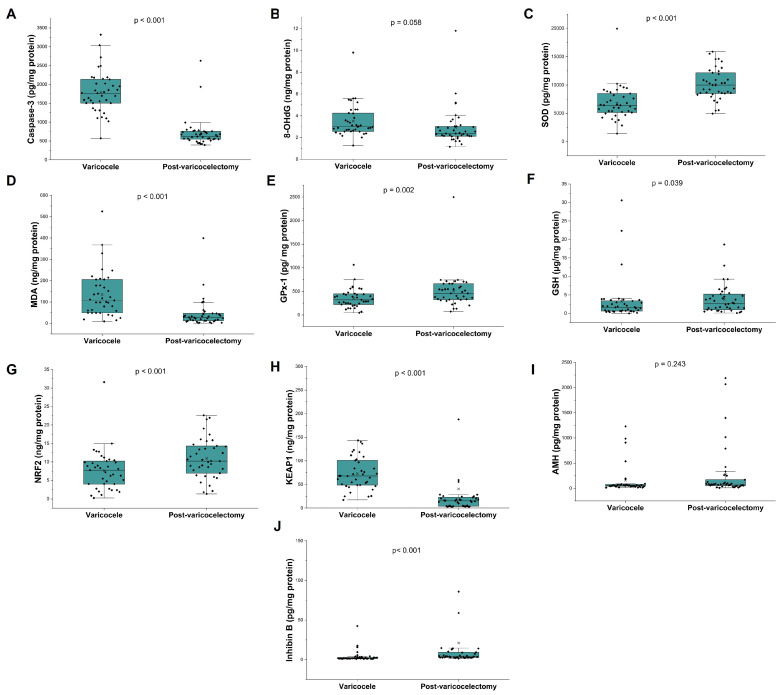
Changes in seminal oxidative stress, antioxidant defense, Sertoli cell markers, and apoptosis after varicocelectomy. Box-and-whisker plots with individual data points showing seminal levels of (**A**) caspase-3, (**B**) 8-OHdG, (**C**) SOD, (**D**) MDA, (**E**) GPx-1, (**F**) GSH, (**G**) NRF2, (**H**) KEAP1, (**I**) AMH, and (**J**) inhibin B in varicocele and post-varicocelectomy samples. Between-group comparisons were performed using the Wilcoxon signed-rank test; *p* values are displayed above each panel.

Postoperative changes in seminal biomarkers are illustrated in Figure 3. Varicocelectomy was associated with a significant reduction in apoptotic activity, as evidenced by decreased caspase-3 levels (*p* < 0.001). Oxidative stress markers also improved, with a significant reduction in MDA (*p* < 0.001) and a borderline decrease in 8-OHdG (*p* = 0.058). Antioxidant defense mechanisms were enhanced following surgery. Significant increases were observed in SOD (*p* < 0.001), GPx-1 (*p* = 0.002), and GSH (*p* = 0.039). Furthermore, redox regulatory signaling shifted toward an antioxidant profile, with NRF2 levels increasing and KEAP1 levels decreasing (both *p* < 0.001). Among Sertoli cell–related markers, inhibin B increased significantly after varicocelectomy (*p* < 0.001), whereas AMH remained unchanged.

### 3.7. Correlations Among Percentage Changes in Biomarkers and Seminal Parameters

**Figure 4:**
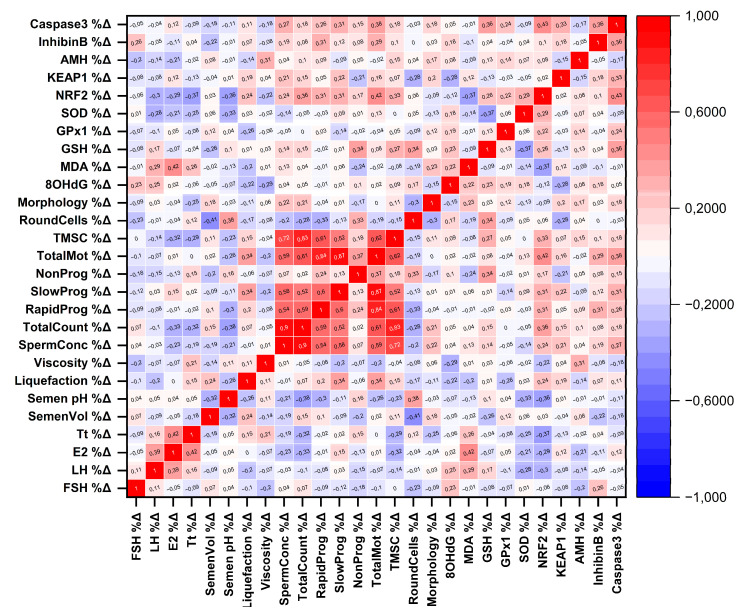
Spearman correlation matrix of pre–post percentage changes (%Δ) in clinical, semen, and biomarker parameters. Correlation heatmap showing Spearman’s rank correlation coefficients (ρ) calculated between percentage changes (%Δ) of all variables from pre- to post-varicocelectomy. Percentage change was computed as %Δ = 100 × (Post − Pre) / Pre for each participant. Cells display ρ values (rounded to two decimals). Red indicates more positive correlations, whereas blue indicates more negative correlations; greater color intensity reflects stronger correlation strength.

Spearman correlation analysis of percentage changes (Δ%) following varicocelectomy revealed several significant associations between biomarker dynamics and reproductive parameters (Figure 4). Increases in NRF2 were positively correlated with improvements in total sperm count (ρ = 0.36, *p* = 0.024), total motility (ρ = 0.42, *p* = 0.009), and total motile sperm count (ρ = 0.33, *p* = 0.042). Conversely, ΔNRF2 showed inverse correlations with changes in total testosterone (ρ = −0.37, *p* = 0.020) and semen pH (ρ = −0.36, *p* = 0.024). Changes in caspase-3 were positively correlated with changes in total motility (ρ = 0.38, *p* = 0.019), while ΔMDA was positively associated with Δestradiol levels (ρ = 0.42, *p* = 0.008). Additionally, ΔGSH correlated positively with changes in non-progressive motility (ρ = 0.34, *p* = 0.037) and round cell count (ρ = 0.34, *p* = 0.032), whereas ΔSOD showed an inverse correlation with changes in semen pH (ρ = −0.33, *p* = 0.037).

## 4. Discussion

The present study yielded several important findings. First, compared with normozoospermic controls, patients with varicocele exhibited markedly impaired semen parameters and significantly reduced bilateral testicular volumes. In addition, varicocele was associated with a distinct seminal molecular profile characterized by increased oxidative DNA damage, reflected by elevated levels of 8-OHdG, reduced antioxidant defense as indicated by decreased SOD levels, and enhanced apoptotic signaling demonstrated by increased caspase-3 levels. Second, varicocelectomy was associated with significant improvements in semen quality accompanied by a reduction in oxidative stress markers, including a significant decrease in MDA levels and a borderline reduction in 8-OHdG. In parallel, antioxidant defense mechanisms were strengthened following surgery, as evidenced by increased levels of SOD, GPx-1, and reduced GSH. Notably, postoperative changes also suggested a restoration of redox homeostasis through activation of the NRF2 signaling pathway, accompanied by a reduction in KEAP1 levels.

Furthermore, among Sertoli cell–related biomarkers, seminal inhibin B levels were significantly reduced in patients with varicocele but increased markedly following varicocelectomy, suggesting partial recovery of Sertoli cell function after surgical correction. In contrast, seminal AMH levels did not show significant differences either between groups or after surgery.

Consistent with previous studies, our results demonstrate increased oxidative DNA damage and reduced antioxidant defense in patients with varicocele, reflected by higher 8-OHdG levels and decreased SOD levels. Several earlier investigations have similarly reported elevated ROS levels, increased lipid peroxidation, and reduced antioxidant capacity in the semen of men with varicocele, supporting the central role of oxidative stress in the pathogenesis of varicocele-related infertility [3,6,7]. Interestingly, although some previous studies have reported markedly increased lipid peroxidation markers in varicocele patients, MDA levels in our cohort did not differ significantly between groups at baseline. This pattern may reflect the context-dependent nature of lipid peroxidation in varicocele. Barradas et al. reported that seminal oxidative stress was elevated mainly in adolescents with varicocele and impaired semen quality, whereas those with varicocele but preserved semen parameters showed no significant difference from controls [29]. However, a significant reduction in MDA levels was observed following varicocelectomy in the present study, suggesting that surgical correction may effectively attenuate oxidative stress-mediated damage. These findings are consistent with the study by Ni et al., which likewise demonstrated a significant postoperative decrease in seminal MDA levels [30]. In parallel, the postoperative increases observed in key components of the antioxidant defense system, including SOD, GPx-1, and GSH, indicate an enhancement of seminal antioxidant capacity after surgery. Collectively, these findings suggest that oxidative DNA damage and apoptotic activation may represent earlier or more sensitive indicators of varicocele-related cellular injury than lipid peroxidation. At the same time, varicocelectomy appears capable of partially restoring the oxidative–antioxidant balance and preserving sperm DNA integrity. This interpretation is further supported by Blumer et al., who reported increased sperm DNA fragmentation and impaired sperm function in men with varicocele despite unchanged lipid peroxidation levels [31]. Moreover, some studies have found no significant differences between varicocele patients and normozoospermic controls in certain oxidative stress markers, including MDA and GPx [32]. Furthermore, the composition of the control group may have contributed to the absence of significant baseline differences in certain biomarkers. As noted in the limitations section, normo-zoospermic men without confirmed natural fertility were used as controls, and it is well recognized that a substantial proportion of normozoospermic individuals may harbor elevated seminal oxidative stress despite normal semen parameters [33], which could have attenuated the magnitude of between-group differences observed in the present study.

Among the molecular pathways regulating cellular antioxidant defense, the NRF2–KEAP1 signaling axis plays a central role in maintaining redox homeostasis [15]. Under basal conditions, KEAP1 binds NRF2 in the cytoplasm and promotes its ubiquitination and degradation, whereas oxidative stress disrupts this interaction, allowing NRF2 to translocate to the nucleus and activate antioxidant response elements that regulate genes involved in antioxidant defense, including SOD, catalase, and GPx [34]. These enzymes are essential for maintaining redox homeostasis during spermatogenesis. In varicocele, persistent redox imbalance may impair this adaptive system [35]. In the present study, the increase in NRF2 levels together with the decrease in KEAP1 levels following varicocelectomy suggests that surgical correction may contribute to the restoration of redox homeostasis through activation of NRF2-mediated antioxidant defense mechanisms. In addition, the positive association between postoperative changes in NRF2 levels and improvements in sperm count and motility further supports a potential link between redox regulatory mechanisms and spermatogenic function. Nevertheless, because the NRF2–KEAP1 pathway activity was not directly evaluated at the molecular level, further studies are required to clarify this relationship mechanistically.

Apoptosis represents an important mechanism linking varicocele-associated oxidative stress to impaired spermatogenesis. Although apoptosis normally functions as a physiological regulatory process that maintains germ cell homeostasis during spermatogenesis, excessive apoptotic activity has been frequently reported in infertile men and in testicular tissue affected by varicocele [36,37]. Testicular hypoxia, a key pathophysiological feature of varicocele, has been shown to increase the expression of hypoxia-inducible factor-1α (HIF-1α) and promote apoptosis in spermatogenic cells. At the molecular level, both intrinsic and extrinsic apoptotic pathways converge on the activation of caspase-3, a central executioner of apoptosis that has been associated with increased ROS levels in semen [37]. Previous studies have demonstrated elevated apoptotic markers, including caspase-3, in the testes and semen of patients with varicocele, while varicocelectomy has been reported to attenuate these alterations and suppress apoptosis [38]. Consistent with these findings, our results showed significantly higher seminal caspase-3 levels in the varicocele group, suggesting increased apoptotic activity likely driven by oxidative stress-related DNA damage. Notably, the significant reduction in caspase-3 levels after varicocelectomy further supports the hypothesis that surgical correction may alleviate ROS-mediated apoptotic signaling and contribute to the restoration of spermatogenic function.

Inhibin-B is a glycoprotein hormone produced by Sertoli cells and secreted primarily into the seminiferous tubule lumen from the apical surface of these cells [39]. Because inhibin-B can be present at higher concentrations in seminal plasma than in serum, seminal levels may represent a more direct indicator of spermatogenesis and Sertoli cell function. In the literature, inhibin-B has predominantly been evaluated in serum and widely investigated in relation to spermatogenic activity and semen parameters [40,41]. In the present study, seminal inhibin-B levels were significantly lower in patients with varicocele compared with normozoospermic controls, whereas a significant increase was observed following varicocelectomy. These findings are consistent with previous reports demonstrating reduced seminal inhibin-B levels in infertile men with varicocele and an increase following treatment of varicocele [42]. In addition, the high AUC values obtained for inhibin-B in our analysis suggest that this marker may reflect varicocele-associated cellular damage and alterations in spermatogenic function.

Despite the strengths of the present study, several limitations should be acknowledged. First, the study was conducted at a single center with a relatively limited sample size, which may restrict the generalizability of the findings. Second, biomarker measurements were performed in seminal plasma, which may not fully reflect molecular alterations occurring within the testicular microenvironment. Therefore, causal relationships between the biomarkers cannot be definitively established. Direct verification of intracellular molecular interactions such as NRF2 nuclear translocation, KEAP1-NRF2 binding, or caspase-3 activation state would require complementary approaches including co-immunoprecipitation, ChIP assay, or pathway reporter systems, which were beyond the scope of the present study. In addition, the cut-off values obtained from ROC analyses were not validated in independent patient cohorts, and therefore the clinical applicability of these biomarkers requires confirmation in larger and multicenter studies. Third, the postoperative follow-up period was limited to 6 months. Although this interval encompasses two complete spermatogenic cycles and captures the primary window of semen parameter improvement following varicocelectomy, it remains possible that molecular biomarkers such as 8-OHdG and AMH may exhibit further changes with prolonged follow-up. Long-term changes in these markers, as well as their relationship with fertility outcomes such as natural pregnancy rates, were not evaluated and warrant investigation in future studies. Fourth, the normozoospermic control group was defined on the basis of semen parameters meeting the WHO 2021 lower reference limits, without confirmed natural fertility. Because some men with normozoospermic semen profiles may still exhibit elevated seminal oxidative stress despite normal conventional semen parameters, subclinical redox imbalance cannot be fully excluded in the control group. This may have attenuated the magnitude of between-group differences observed for certain biomarkers in the present study. Future studies should ideally include proven fertile men as controls to more accurately characterize the redox profile associated with varicocele-related infertility. Nevertheless, the strengths of this study include the presence of a normozoospermic control group, the simultaneous evaluation of multiple biomarkers reflecting oxidative stress, apoptotic activity, and Sertoli cell function, and the analysis of postoperative changes within the same patient cohort.

## 5. Conclusions

In conclusion, the present study demonstrates that varicocele is associated with increased oxidative stress, impaired antioxidant defense, and enhanced apoptotic activity in seminal plasma, together with alterations in markers of Sertoli cell function. Varicocelectomy was associated with a reduction in oxidative stress markers, and strengthening of antioxidant defense mechanisms. In addition, the postoperative increase in NRF2 levels accompanied by a decrease in KEAP1 levels suggests that restoration of redox homeostasis after surgical correction may involve activation of NRF2-mediated antioxidant pathways. The concomitant reduction in caspase-3 levels further supports the concept that modulation of oxidative stress may attenuate apoptosis-related pathways in varicocele. Understanding the molecular pathways underlying these improvements may contribute to the development of targeted therapeutic strategies for oxidative stress-related male infertility.

## Figures and Tables

**Table 1:** Baseline demographic and clinical characteristics of normozoospermic controls and patients with varicocele.

	Normozoospermic (*n* = 44) Median (Q1–Q3) or (Mean ± SD)	Varicocele (*n* = 39) Median (Q1–Q3) or (Mean ± SD)	*p*-Value
Age (years)	29 (26–38.8)	28 (24–30)	0.021 ^#^
Height (cm)	175.5 ± 6.49	178.7 ± 6.01	0.025 *
Weight (kg)	82 (74.3–90.8)	76 (70–95)	0.612 ^#^
BMI (kg/m^2^)	26.4 (24.4–29.8)	24.9 (21.6–29.4)	0.167 ^#^
Waist circumference (cm)	95 (91–105)	88 (85–102)	0.049 ^#^
Total cholesterol (mg/dL)	182.0 (161.3–203.5)	188.0 (161.0–216.0)	0.606 ^#^
HDL (mg/dL)	44.9 (38.8–55.2)	42.2 (38.5–52.9)	0.635 ^#^
LDL (mg/dL)	111.95 ± 34.81	115.93 ± 33.83	0.600 *
Triglyceride (mg/dL)	103.5 (75.3–157.0)	101.0 (72.0–167.0)	0.862 ^#^
Glucose (mg/dL)	90.5 (86.3–100.0)	89.0 (82.0–93.0)	0.069 ^#^
Creatinine (mg/dL)	0.91 ± 0.11	0.92 ± 0.12	0.734 *
CRP (mg/L)	1.10 (0.62–2.40)	1.34 (0.58–4.54)	0.172 ^#^
WBC (×10^3^/µL)	6.83 ± 1.35	7.33 ± 2.03	0.188 *
Hemoglobin (g/dL)	15.45 (14.40–15.98)	15.50 (14.60–15.80)	0.931 ^#^
Neutrophil (×10^3^/µL)	3.87 (2.80–4.89)	3.69 (2.86–5.31)	0.678 ^#^
Lymphocyte (×10^3^/µL)	2.12 (1.79–2.76)	2.51 (1.96–2.83)	0.333 ^#^
RDW (fL)	44.15 (42.78–45.55)	44.90 (43.40–46.30)	0.183 ^#^
Platelet (×10^3^/µL)	251.16 ± 59.03	239.23 ± 47.79	0.319 *

Data are presented as mean ± SD for normally distributed variables and median (Q1–Q3) for non-normally distributed variables. Between-group comparisons were performed using the independent samples *t*-test (*) for normally distributed variables and the Mann–Whitney U test (^#^) for non-normally distributed variables. A *p*-value < 0.05 was considered statistically significant.

**Table 2:** Hormonal, testicular volume, and semen parameters in normozoospermic controls and patients with varicocele.

	Normozoospermic (*n* = 44) Median (Q1–Q3) or (Mean ± SD)	Varicocele (*n* = 39) Median (Q1–Q3) or (Mean ± SD)	*p*-Value
Right testis volume (mL)	20.45 (18.17–22.20)	15.50 (12.00–18.00)	<0.001 ^#^
Left testis volume (mL)	21.50 (20.00–37.60)	13.00 (9.50–16.10)	<0.001 ^#^
Total testicular volume (mL)	42.75 (39.60–84.30)	29.00 (21.00–33.60)	<0.001 ^#^
Total testosterone (ng/dL)	447.8 ± 169.5	486.3 ± 191.8	0.334 *
Estradiol (pg/mL)	28.90 (23.36–36.06)	27.87 (23.00–32.07)	0.425 ^#^
Semen volume (mL)	4.25 (3.02–4.97)	3.36 (2.55–4.56)	0.054 ^#^
Semen pH	7.60 (7.40–7.80)	7.60 (7.40–7.80)	0.607 ^#^
Liquefaction time (min)	37.50 (30.00–50.00)	30.00 (25.00–40.00)	0.049 ^#^
Sperm concentration (×10^6^/mL)	58.50 (38.13–89.75)	8.30 (1.50–15.90)	<0.001 ^#^
Total sperm count (×10^6^)	216.00 (143.58–361.07)	24.90 (5.66–53.28)	<0.001 ^#^
Total motile sperm count (×10^6^)	94.19 (51.72–187.06)	6.89 (1.03–19.69)	<0.001 ^#^
Total motility (%)	44.64 ± 7.44	27.18 ± 10.86	<0.001 *
Progressive motility (%)	37.75 ± 7.21	22.15 ± 9.84	<0.001 *
Rapid progressive (%)	16.00 (13.25–18.00)	8.00 (3.00–10.00)	<0.001 ^#^
Slow progressive (%)	22.11 ± 3.66	15.23 ± 6.06	<0.001 *
Non-progressive (%)	6.00 (5.00–8.00)	5.00 (4.00–6.00)	0.001 ^#^
Morphology (%)	3.00 (2.00–4.00)	0.00 (0.00–1.00)	<0.001 ^#^
Round cell count (×10^6^/mL)	0.55 (0.20–1.00)	0.40 (0.20–0.60)	0.121 ^#^
Viscosity (score)	1 (1–1)	1 (1–1)	0.190 ^#^

Data are presented as mean ± SD for normally distributed variables and median (Q1–Q3) for non-normally distributed variables. Between-group comparisons were performed using the independent samples *t*-test (*) for normally distributed variables and the Mann–Whitney U test (^#^) for non-normally distributed variables. A *p*-value < 0.05 was considered statistically significant.

**Table 3:** Changes in hormonal and semen parameters after varicocelectomy.

	Varicocele Median (Q1–Q3) or (Mean ± SD)	Post-Varicocelectomy Median (Q1–Q3) or (Mean ± SD)	*p*-Value
FSH (IU/L)	5.25 (3.73–7.27)	5.10 (3.22–7.01)	0.675 ^#^
LH (IU/L)	3.91 (3.45–5.61)	4.09 (3.02–5.74)	0.883 ^#^
Estradiol (pg/mL)	27.87 (23.00–32.07)	30.23 (25.00–36.00)	0.182 ^#^
Total testosterone (ng/dL)	499.1 ± 210.6	477.0 ± 208.0	0.629 *
Semen volume (mL)	3.36 (2.55–4.56)	3.48 (2.68–4.45)	0.192 ^#^
Semen pH	7.61 ± 0.31	7.62 ± 0.23	0.818 *
Liquefaction time (min)	30 (25–40)	30 (25–35)	0.254 ^#^
Viscosity (score)	1 (1–1)	1 (1–1)	0.346 ^#^
Sperm concentration (×10^6^/mL)	8.30 (1.50–15.90)	24.00 (6.80–42.00)	<0.001 ^#^
Total sperm count (×10^6^)	24.90 (5.65–53.28)	69.50 (23.64–145.32)	<0.001 ^#^
Rapid progressive motility (%)	8 (3–10)	13 (12–15)	<0.001 ^#^
Slow progressive motility (%)	16 (10–20)	19 (17–21)	<0.001 ^#^
Non-progressive motility (%)	5 (4–6)	5 (5–6)	0.060 ^#^
Total motility (%)	30 (18–36)	39 (35–42)	<0.001 ^#^
Total motile sperm count (×10^6^)	6.89 (1.03–19.69)	26.81 (8.51–72.94)	<0.001 ^#^
Round cell count (×10^6^/mL)	0.40 (0.20–0.60)	0.20 (0.10–0.30)	<0.001 ^#^
Morphology (%)	0 (0–1)	2 (0–4)	<0.001 ^#^

Data are presented as mean ± SD for normally distributed variables and median (Q1–Q3) for non-normally distributed variables. Comparisons between preoperative and postoperative values were performed using the paired samples *t*-test (*) for normally distributed variables and the Wilcoxon signed-rank test (^#^) for non-normally distributed variables. A *p*-value < 0.05 was considered statistically significant.

## Data Availability

The original contributions presented in this study are included in the article; further inquiries can be directed to the corresponding author.
